# Metabolic cofactors NADH and FAD act as non-canonical initiating substrates for a primase and affect replication primer processing *in vitro*

**DOI:** 10.1093/nar/gkaa447

**Published:** 2020-05-28

**Authors:** Christina Julius, Paula S Salgado, Yulia Yuzenkova

**Affiliations:** Centre for Bacterial Cell Biology, Biosciences Institute, Newcastle upon Tyne, NE2 4AX, UK; Centre for Bacterial Cell Biology, Biosciences Institute, Newcastle upon Tyne, NE2 4AX, UK; Centre for Bacterial Cell Biology, Biosciences Institute, Newcastle upon Tyne, NE2 4AX, UK

## Abstract

To initiate replication on a double-stranded DNA *de novo*, all organisms require primase, an RNA polymerase making short RNA primers which are then extended by DNA polymerases. Here, we show that primase can use metabolic cofactors as initiating substrates, instead of its canonical substrate ATP. DnaG primase of *Escherichia coli* initiates synthesis of RNA with NADH (the reduced form of nicotinamide adenine dinucleotide) and FAD (flavin adenine dinucleotide) *in vitro*. These cofactors consist of an ADP core covalently bound to extra moieties. The ADP component of these metabolites base-pairs with the DNA template and provides a 3′-OH group for RNA extension. The additional cofactors moieties apparently contact the ‘basic ridge’ domain of DnaG, but not the DNA template base at the –1 position. ppGpp, the starvation response regulator, strongly inhibits the initiation with cofactors, hypothetically due to competition for overlapping binding sites. Efficient RNA primer processing is a prerequisite for Okazaki fragments maturation, and we find that the efficiency of primer processing by DNA polymerase I *in vitro* is specifically affected by the cofactors on its 5′-end. Together these results indicate that utilization of cofactors as substrates by primase may influence regulation of replication initiation and Okazaki fragments processing.

## INTRODUCTION

Replicative DNA polymerases (DNAPs) are unable to initiate synthesis of DNA *de novo*, i.e. without a primer. Therefore, all organisms and some viruses employ a primase, a specialized RNA polymerase (RNAP) that synthesizes short RNAs which DNAPs can then extend (1–3). The *Escherichia coli* genome is a double-stranded 4.7 Mb DNA. Since the main replicative DNAP, DNA Polymerase III (Pol III), synthesizes DNA in 5′−3′ direction, only one strand (leading) can be synthesized continuously, whereas another strand (lagging) has to be synthesized initially in discrete 1–2 kb long Okazaki fragments. Therefore, while leading strand synthesis involves very few priming events, each Okazaki fragment must start with an RNA primer that is synthesized by the primase DnaG. To switch synthesis from RNA to DNA, primase is displaced and the primer is elongated by Pol III (4). Before the Okazaki fragments are ligated to complete genome replication, the RNA primers need to be removed via the combined actions of DNA polymerase I (Pol I) and/or RNaseH. Pol I acts as an exonuclease, degrading RNA in a 5′→ 3′ direction, and as a DNAP, simultaneously extending the previous Okazaki fragment in a 3′→ 5′ direction (5,6). Replication priming requires a single stranded binding protein, SSB. Primase acts in concert with a number of replication proteins, including helicase DnaB, forming a primosome. At the replication origin the primosome contains additional proteins such as DnaA (7). If replicative DNAP runs into physical obstacles or DNA breaks, the replication fork may collapse (8). To rescue the fork, replication is restarted downstream by re-priming DNA synthesis (9,10). Overall, synthesis of an RNA primer by primase is believed to be a rate limiting step of replication (11), tightly coupled to other steps of the replication process. Thus, primase plays a key role during assembly of the replisome (12), regulation of replication elongation and the Okazaki fragments’ length in both bacterial and eukaryotic systems (11,13,14). In bacteria, primase plays an additional role during the global stress response to starvation via alarmone ppGpp (guanosine tetraphosphate). An amino acid deficit activates synthesis of ppGpp by a ribosome-associated synthase. Resulting quick accumulation of ppGpp in the cell inhibits both transcription and replication. Replication is inhibited at the initiation stage by direct binding of ppGpp at the active site of the DnaG primase (15).

In *E. coli*, DnaG primase recognises a consensus 3′-GTC-5′ motif in DNA and makes a 10–12 nucleotide long RNA primer, starting synthesis with ATP opposite the central thymine of this element (3). The DnaG active site possesses a TOPRIM (Topoisomerase/Primase) domain where three Mg^2+^ ions are coordinated by conserved acidic amino acid residues. For efficient catalysis of the first phosphodiester bond, the initiating ATP is positioned within a pocket between catalytic Mg^2+^ ions and the ‘basic ridge’ region, containing several conserved basic amino acid residues (15). It was suggested that ppGpp binds to an overlapping site within the primase, hence competing with the initiating ATP to inhibit DnaG during starvation (15).

NTP substrates of RNAPs are not the only abundant nucleotides in the cell, another group are cofactors, such as NAD^+^/NADH, FAD and DP-CoA (dephospho-Coenzyme A). These molecules can replace ATP in initiation of RNA synthesis by base-pairing with the template DNA using their ADP part and providing an accessible 3′ hydroxyl group for RNA extension. Taking into account its strong preference for ATP as a first nucleotide, primase could potentially utilize them as initiating substrates. RNAPs of transcription (unrelated to primases (16)) were recently shown to incorporate and retain ADP-containing cofactors NAD^+^/NADH, DP-CoA and FAD at the 5′ end of RNA (17–19) during initiation. This process is template-dependent, i.e. only happens during initiation at promoters coding for ATP as an initiating substrate (20,21). As a result of this non-canonical initiation, some RNA species in bacteria and eukaryotes bear an extra moiety on the 5′ terminus, superficially similar to the eukaryotic cap. RNA species bearing 5′ NAD^+^/NADH cofactors are processed by hydrolases of the NUDIX family, e.g. in *E. coli* by NudC (NADH pyrophosphohydrolase) which generates a 5′ monophosphorylated RNA species that are quickly degraded in the cell (22).

In the present study we sought to address two questions: i) whether primase could use cofactors as the initiating nucleotide, and if the answer is yes, ii) what potential physiological consequences this non-canonical initiation can cause. We found that *E. coli* DnaG primase is capable of initiating RNA synthesis using a number of ADP-containing cofactors *in vitro*, including NADH, FAD and DP-CoA, but not NAD^+^. This reaction requires amino acid residues of the DnaG ‘basic ridge’ region and is inhibited by the global starvation alarmone ppGpp. We also show that cofactors on the 5′-end of an RNA primer specifically and differentially affect its processing by DNA Pol I.

## MATERIALS AND METHODS

### Nucleotides

ATP, GTP and UTP were from GE Healthcare; AMP, ADP, NAD^+^, NADH and FAD, were from Sigma Aldrich, ppGpp from Tebu-Bio.

### Cloning, proteins expression and purification

The original source of *dnaG* and *dnaB* genes were the corresponding pCA24N overexpression vectors from the ASKA collection. For subsequent expression and isolation *dnaG* and *dnaB* genes were transferred to plasmid pET28a. This pET28a-*dnaG* WT (wild type) plasmid was used to generate DnaG mutants with amino acid substitutions K229A, Y230A, K241A and D309A by QuickChange mutagenesis kit and protocol (Agilent).

Plasmid –encoded wild type and mutant C-terminally His_6_-tagged DnaG, DnaB and NudC were expressed in *E. coli* T7 express strain (New England Biolabs). Cells were grown to an OD_600_ = 0.5 at 37°C before induction with IPTG (1 mM), and the growth was continued at ambient temperature overnight. Cells were lysed by sonication in grinding buffer (20 mM Tris–HCl pH 7.9, 200 mM NaCl, 5% glycerol). Proteins were purified by affinity chromatography on Ni-NTA-sepharose (HisTrap, GE Healthcare) in 20 mM Tris–HCl pH 8.0, 600 mM NaCl, 5% glycerol buffer. Cell lysate clarified by centrifugation was loaded onto the column in the presence of 10 mM imidazol to reduce non-specific protein binding. The column was washed with buffer containing 25 mM imidazole, and bound proteins eluted with buffer containig 200 mM imidazole. Fractions containing target proteins were pooled and further purified by ion exchange chromatography on ResourseQ column (GE Healthcare). The pure protein fractions were pooled and dialysed against storage buffer (20 mM Tris–HCl pH 8, 50% glycerol, 200 mM KCl, 0.5 mM EDTA, 1 mM DTT).

### *In vitro* primer synthesis

RNAs were synthesized by DnaG primase (1 μM) on single stranded 5′- biotynilated DNA template with the sequence 5′-bio-CGGACACACACACACTGCGAAGC; or hairpin template for subsequent Pol I primer degradation experiments 5′- bio-TTTACGCTTCGTTGACACACACACTGCGCGTTTGGGAAAACTCTTTCCCAAAC. For the experiments with DnaG mutants and for Pol I degradation, the primer was synthesized at the presence of DnaB helicase (3 μM).

Reactions contained 500 μM ATP or cofactors, 100 μM UTP and 10 μM [α^32^P]-GTP 5 Ci/mmol (Hartmann Analytic) in primase reaction buffer (50 mM HEPES pH 7.0, 20 mM Mg-acetate, 100 mM K-glutamate, 10 mM DTT) at 30°C for 10 min, unless otherwise indicated in the Results section. The reactions were stopped by addition of formamide-containing loading buffer (85% formamide, 1× Tris–borate EDTA buffer, 7 M urea, 20 mM EDTA, 100 ug/ml heparin, 0.02% bromophenol blue, 0.02% xylene cyanol). Products were separated on denaturing polyacrylamide gels (20% acrylamide, 3% bis-acrylamide, 7 M urea, 1× Tris–borate EDTA buffer), revealed by PhosphorImaging (GE Healthcare), and analysed using ImageQuant software (GE Healthcare).

Hydrolysis of the 5′ NADH-RNA by NudC was performed by addition of the 100 nM of purified protein in primase reaction buffer and incubation at 30°C for 10 min.

For apparent *K*_M_ determination initiating substrates were used in concentrations ranging from 5 μM to 1 mM; time intervals were 5, 10, 20, 30, 60, 120, 300 s. For each of the concentrations, reaction rates were determined by fitting data into exponential equation using SigmaPlot software. These parameters were fitted into Michaelis-Menten equation *V* = *V*_max_ × [*S*]/(*K*_m_ + [*S*]) using non-linear regression in SigmaPlot software to produce *K*_M_ values.

### ppGpp competition assay

ppGpp at final concentrations of 100 μM, 600 μM and 1 mM was added to the primase reaction prior the initiating substrates. Initiating substrates were used at 100, 600 and 1 mM concentrations, to generate relative rates of primer synthesis for plots on the Figure 4A–C. The end points of these curves were used to generate residual activity values, presented on Figure 4D as a percentage of the activity without ppGpp.

### DNA Polymerase I (Pol I) – dependent primer degradation assay

Primer was generated on the hairpin template by DnaG (1 μM) and DnaB (3 μM) premixed at ambient temperature with initiating substrate (100 μM ATP or 500 μM of NADH, FAD, DP-CoA), 10 μM [α^32^P]-GTP 5 Ci/mmol, 100 μM UTP, in primase reaction buffer (50 mM HEPES pH 7.5, 20 mM Mg-acetate, 100 mM K-glutamate, 10 mM DTT) at 30°C for 10 min. The reaction was stopped by adding a final concentration of 1 M NaCl, and proteins were removed from reaction by binding to Ni-NTA agarose beads for 5 min at ambient temperature, and pelleting the beads by low speed centrifugation. The supernatant was transferred to a gel filtration column (Micro-Bio Spin 6, BioRad). DNA–RNA hybrid was immobilized on streptavidine beads via biotin on the DNA template, and non-bound material removed by washing with PolI buffer (20 mM Tris–HCl pH 8.0, 50 mM KCl, 10 mM MgCl_2_). Pol I (*E. coli* source, Fisher Scientific) was added at 0.25 U/μl, alongside 10 μM dNTPs. Reactions were incubated at 37°C and stopped after 5, 10, 20, 30, 45, 60, 120 and 180 s with formamide-containing loading buffer as above. Products were separated on the denaturing 23% polyacrylamide gel, revealed by PhosphorImaging (GE Healthcare), and bands’ intensities quantified using ImageQuant software (GE Healthcare). The percentage of the initial RNA13 band, compared to the total radioactivity in the lane, was plotted against time to generate plots on Figure 5.

### Structural modelling of the DnaG complexes with non-canonical substrates and ppGpp

Structural models of *E. coli* primase (PDB ID 1DDE) and *Staphylococcus aureus* primase in complex with ATP and ppGpp (PDB IDs 4EDG and 4EDT, respectively) were superimposed using the structure visualization and model building tool COOT (23). The protein models superimpose with an overall RMSD (root mean square deviation) of 2.8 and 2.7 Å, respectively. Binding of FAD and NADH was modelled using COOT ligand library to generate the non-canonical initiation ligands. The ligand models were superimposed on the ATP and ppGpp bound *S. aureus* models using the ligand superposition tools in COOT. In *S. aureus* DnaG-ATP complex, the nucleotide has two possible conformations. For superposition of FAD and NADH, the conformation common to complexes of *S. aureus* with the other nucleic acid nucleotides (GTP, CTP, UTP) was used. Fitting was optimised to match the phosphate, sugar and base moieties in each ligand with those in the ATP bound form of *S. aureus* primase using angle editing tools, followed by geometry optimisation within COOT. The remaining regions of each ligand were modelled such as any clashes with surrounding residues were minimised. ppGpp was modelled into the *E. coli* primase structure based on the superposition of the protein models, followed by geometry optimisation in COOT to avoid clashes with surrounding residues.

## RESULTS

### *E. coli* DnaG primase initiates RNA primer synthesis using NADH, FAD and DP-CoA, but not NAD^+^

**Figure 1.**
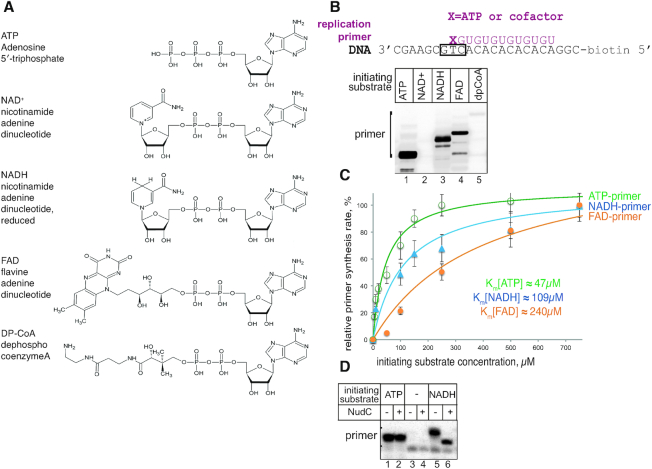
Primase DnaG initiates replication primer synthesis with NAD^+^, NADH, FAD and DP-CoA. (**A**) Structures of cofactor molecules (NAD^+^, NADH, FAD and DP-CoA) in comparison to ATP, the preferred initiating substrate of primase. (**B**) Replication primer synthesis on single-stranded DNA template, scheme above, with ATP, NAD^+^, NADH, FAD and DP-CoA as initiation substrates. (**C**) Plots of the dependencies of the primer amount from concentrations of the initiating ATP, NADH and FAD. Solid lines represent the graphical fits of data (using SigmaPlot software) to the Michaelis–Menten equation. Error bars represent standard deviations from triplicate experiments. (**D**) RNA primer with 5′-NADH is susceptible to cleavage by NudC hydrolase after DnaG is removed from the complex with high salt wash. Note that absence of ATP from the reaction results in an increased amount of non-specific product, which is not susceptible to NudC in lanes 5, 6. We assume that this band results from initiation with GTP present in the reaction.

DnaG primase functions as a low-processivity RNA polymerase able to start *de novo* RNA synthesis on DNA. We wanted to test if primase can initiate synthesis using ADP- containing metabolic cofactors (structures on Figure 1A), by analogy with RNA polymerases of transcription.

We used a general priming system, i.e. a minimal system in the absence of single-strand DNA-binding protein (24). This set-up requires only DnaG for RNA primer synthesis on short single-stranded DNA template containing a single 3′-GTC-5′ recognition motif (scheme on Figure 1B) (7). The DNA template we used allows primase to synthesise a 13nt long RNA using a subset of NTPs lacking CTP.

We found that DnaG makes a 13nt long RNA product with either ATP, or NADH, FAD and DP-CoA as initiating substrates, supplemented with GTP and UTP as elongating substrates (Figure 1B) (to avoid confusion, we refer to the length of RNA in the canonical nucleotides, even though the NADH and FAD are dinucleotides). Notably, DnaG incorporates NAD^+^ much less efficiently than NADH, in contrast to other RNAPs, which do not discriminate between NAD^+^ and NADH (21). DP-CoA incorporation was very inefficient, therefore we decided not to investigate NAD^+^ or DP-CoA substrates in subsequent experiments.

To determine if non-canonical initiation would be possible in living cells, we measured apparent Michaelis constants for ATP and the cofactors. We found that for ATP, NADH and FAD as initiating substrates the constants were 46.6, 109 and 240 μM, respectively (Figure 1C). These values for cofactors are in the range of their cellular concentrations, suggesting that non-canonical initiation is a physiologically relevant reaction (25) (see also Discussion).

It was previously shown that the NudC hydrolase of *E. coli* cleaves the pyrophosphate bond of the 5′-NADH, producing nicotinamide mononucleotide (NMN) and monophosphorylated RNA species. We found that after synthesis by DnaG, the 5′-NADH of the primers was susceptible to NudC activity (Figure 1D). Interestingly, hydrolysis by NudC was only observed after primase was removed from the immobilized nucleic acid complex using a high salt wash. This result is in agreement with the view that full length primer stays bound to the DNA template and in complex with primase (26).

### Initiating ADP-cofactors do not affect DnaG specificity of initiation

**Figure 2.**
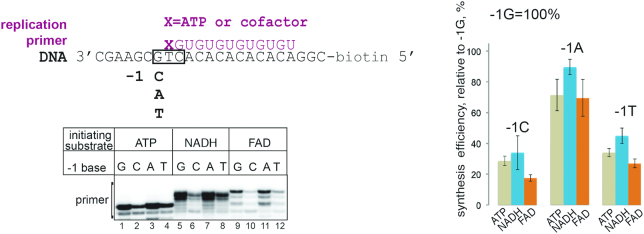
Cofactors do not make specific contacts with the -1 DNA template base. Synthesis efficiency of RNA13 started with ATP, NADH or FAD on DNA templates with C, A or T at position –1 was compared to the consensus –1G template. Relative efficiency of synthesis is shown in percentage from efficiency of the –1G template, error bars reflect standard deviations from three independent experiments.

Extra moieties of cofactors could make additional contacts with either the DNA template upstream of the +1 start site, or the DnaG protein. We hypothesized that such additional contacts with DNA might influence the initiation specificity of DnaG. The identity of the base at the –1 DNA template position affects the efficiency of non-canonical initiation of transcription by *E. coli* RNAP with NAD^+^ (20). The template we used has a T at +1 position to base-pair with initiating A, and a G at –1 position (scheme on Figure 2), and we will refer to this template as a –1G template. To test if cofactor incorporation is influenced by the identity of the –1 base, we tested synthesis of RNA13 on templates with the –1G changed to the three alternative bases, resulting in –1C, –1A and –1T templates (Figure 2). This experiment was performed with 50 μM ATP and 500 μM cofactors to reflect the differences in their corresponding *K*_M_s. We found that in general DnaG primase preferred purines in this position, and the least preferred base is C (Figure 2). Initiation with cofactors did not change these preferences, suggesting that cofactors do not make specific contacts with the –1 base of the template. This result also implies that cofactors as substrates do not change specificity of DnaG initiation, and presumably do not cause spurious or excessive initiation in the cell.

### Initiation of RNA synthesis with cofactors requires the ‘basic ridge’ amino acid residues of DnaG

**Figure 3.**
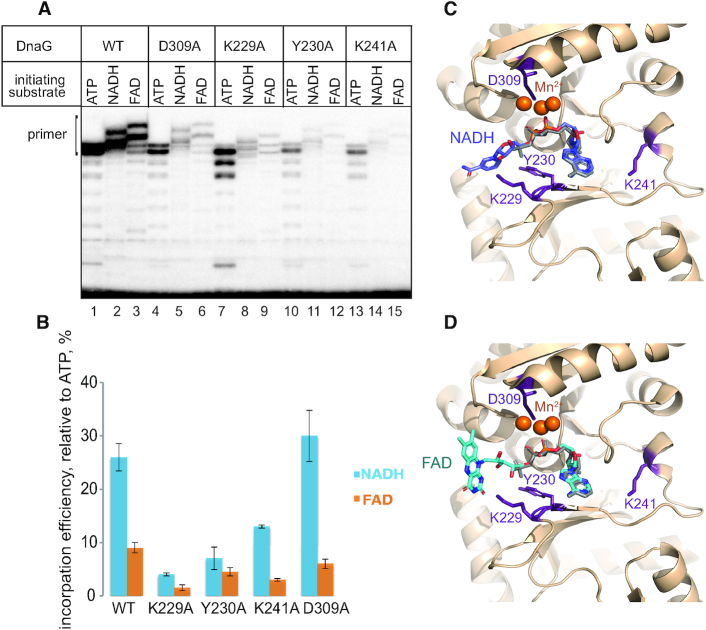
DnaG basic ridge residues affect initiation with NADH and FAD. (**A**) Primer synthesis by WT DnaG and DnaGK229A, DnaGY230A, DnaGK241A, DnaG D309A mutants using ATP, NADH or FAD as a starting nucleotide. (**B**) Relative efficiency of NADH and FAD utilisation as initiation substrates in comparison to that of ATP, for WT DnaG and DnaGK229A, DnaGY230A, DnaGK241A, DnaGD309A mutants. Error bars reflect standard deviations from three independent experiments. (**C** and **D**) Structural models of *E. coli* DnaG complex with NADH and FAD, respectively. NADH is in blue, FAD is in teal, amino acid residues which we tested for efficiency of cofactor initiation are in magenta, orange spheres are catalytic Mn^2+^ ions. For comparison, ATP used to superimpose the non-canonical nucleotides is shown in grey.

Cofactors accommodated in a nucleotide-binding pocket could make additional specific interactions with DnaG, considering their possession of extra moieties. A number of amino acid residues, including several in the ‘basic ridge’ region of primase, were implicated in initiation nucleotide binding, based on sequence conservation amongst primases and structural information for *S. aureus* primase (15). We tested synthesis of a primer by DnaG with amino acid substitutions, K229A, Y230A, K241A (all belonging to the ‘basic ridge’) and D309A (participating in metal chelation, not part of the ‘basic ridge’), which were all previously shown to influence initiating substrate incorporation (1,15), in the presence of DnaB. Using DnaB along with DnaG, we found that ‘basic ridge’ substitutions K229A, Y230A and to some extent K241A specifically inhibited initiation with NADH and FAD (Figure 3 and Supplementary Figure S1). In contrast, D309A demonstrated relative efficiency of substrates utilization close to that for the WT enzyme. We assumed that the NMN and flavin mononucleotide (FMN) moieties of the corresponding cofactors might make contacts with these amino acid residues either during binding of the initiating substrates or during the very first step of RNA synthesis.

In order to investigate if the NMN and FMN moieties could establish extra contacts with the residues in the basic ridge, particularly K229 and Y230, we modelled NADH and FAD to the *E. coli* primase structure (27), using the previously determined ATP-bound forms of *S. aureus* DnaG primase (15). Superposition of the ATP moieties in the nucleotide-binding pocket allow for the NMN and FMN regions of the cofactors to be accommodated in the basic ridge region (Figure 3C, D). Indeed, our models suggest that extensive interactions exist between the cofactors and K229 and Y230 (See Supplemental Table S1 for the distances predicted in our models). These extended contact networks would presumably stabilize non-canonical initiation, making it more prone to be disrupted in the mutants analysed. In our current model, K241 contacts cofactors adenosine via a water molecule (Supplemental Table S1), similarly to the primase-NTP models of *S. aureus*. We hypothesize that this residue might possibly play a role during extension of the RNA, even though current models suggest less direct contacts.

### ppGpp strongly inhibits non-canonical initiation by DnaG

**Figure 4.**
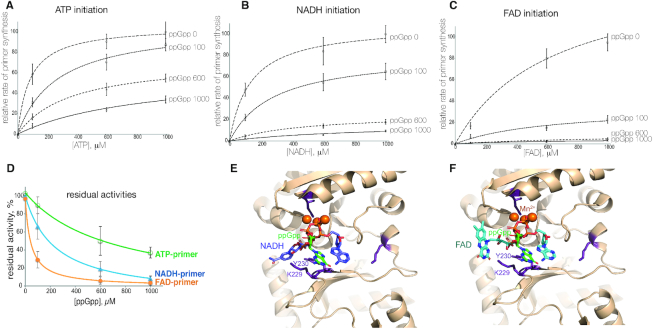
Initiation with cofactors is more susceptible to inhibition by ppGpp than initiation with ATP. (**A–C**) Primer synthesis was measured at increasing concentration of initiation substrate – ATP, NADH and FAD. For each of the initiation substrates (designated on top as ATP initiation, NADH initiation and FAD initiation) an experiment was performed either without ppGpp (curve 0 ppGpp) or with increasing concentration of ppGpp – 100, 600 and 1000 μM. (**D**) Residual DnaG activities at 1000 μM initiation substrate are plotted against an increasing concentrations of ppGpp, in percentages from amounts in the absence of ppGpp. Error bars reflect standard deviations obtained from three independent experiments. (**E** and **F**) Structural models of *E. coli* DnaG complex with NADH and ppGpp and FAD and ppGpp, respectively. ppGpp is in green, NADH is in blue, FAD is in teal, amino acid residues which we tested for efficiency of cofactor initiation are in magenta, orange spheres are catalytic Mn^2+^ ions.

Under nutrient deficient conditions, replication is inhibited via the action of the global stringent response alarmone ppGpp on DnaG. ppGpp binds DnaG near the active site (15), presumably overlapping with the binding site for substrates during the initiation of primer synthesis. To test how efficiently ppGpp inhibits non-canonical initiation, in comparison to canonical initiation with ATP, we measured the maximal rate of RNA product formation in the presence of increasing concentrations of ppGpp at different concentrations of initiating substrates ATP, NADH and FAD (Figure 4A, B, C, respectively). As can be seen from these plots, ppGpp inhibits the reaction more efficiently if it is performed with lower concentrations of substrates, suggesting a competitive mechanism of inhibition. Yet, the mechanism is not purely competitive, since even at highest substrate concentrations ppGpp still inhibited synthesis. It can also be noted that ppGpp competes more efficiently with NADH and especially FAD (Figure 4A–C). Altogether these effects are more apparent from the plot of residual activities at 1 mM concentration of the initiation substrates versus ppGpp concentration on Figure 4D. Based on these data we suggest a mixed type of inhibition of primer synthesis by ppGpp. We further supported these results by structural modelling. As seen in the *S. aureus* primase form bound to ppGpp where it partially overlaps with ATP (15), NADH and FAD would also result in a partial overlap in the nucleotide-binding pocket according to our models (Figure 4E, F). Moreover, the extra interactions with K229 and Y230 seen in these models are also partially overlapped by ppGpp binding (Figure 4E, F), further suggesting a mixed inhibition mechanism. Using the same procedure as described for above to superimpose the ATP moieties in NADH and FAD to ppGpp would result in clashes of the NMN and FMN regions with DnaG protein, suggesting possible different orientations in early binding events of the three molecules.

### 5′- cofactors differentially affect primer processing by Pol I

**Figure 5.**
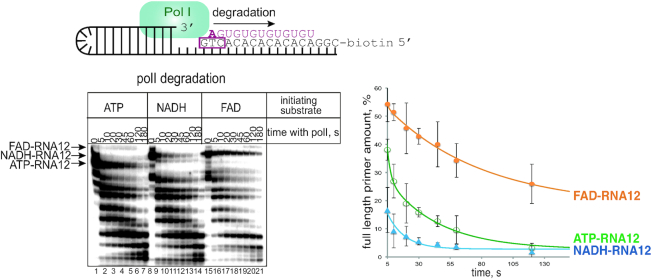
Cofactors at the 5′- end of a primer differentially affect its processing by Pol I - NADH speeds it up, FAD inhibits. Scheme of the hairpin DNA substrate with annealed RNA made by DnaG is shown above the gel. On the gel products of time dependent degradation of the ATP-RNA12, NADH-RNA12 FAD-RNA12 made with either ATP, NADH or FAD as initiating substrates are shown. Below the gel image, the same degradation kinetics are presented on a plot. Plot shows amount of the initial full-length of primer as a function of incubation time with Pol I and dNTPs. Error bars reflect standard deviations obtained from three independent experiments.

To complete replication, the leading strand and Okazaki fragments of a lagging strand need to be processed and ligated. This processing involves RNA primer removal followed by extension of the upstream Okazaki fragment; both of these critical events can be achieved by Pol I which uniquely possesses both 5′→ 3′exonuclease and 3′-DNA polymerase activities. We examined whether the presence of cofactors on the 5′-end of an RNA primer affects its removal by Pol I. This experiment was done using a DNA-RNA scaffold mimicking a replication intermediate (Figure 5, top). The substrates consisted of a hairpin-containing DNA template with an RNA primer produced by DnaG in the presence of the various initiating nucleotides (ATP-RNA12, FAD-RNA12 or NADH-RNA12). The DNA–RNA construct was immobilised on streptavidin beads via biotin on a DNA template, which ensured that any processing observed occurs on an RNA primer annealed to a DNA template. DnaG primase and any free RNA was subsequently removed by washing with high salt containing buffer. Addition of Pol I and dNTPs to this complex led to a stepwise processing of the primer, as seen from the kinetics of RNA degradation on a gel image, and from the plot below (Figure 5). Interestingly, compared to the degradation rate of 5′-ATP-RNA12, Pol I exonuclease activity was stimulated by a 5′-NADH while it was inhibited by a 5′-FAD (Figure 5).

## DISCUSSION

Here we showed that DnaG primase of *E. coli* initiates synthesis of a replication primer with ADP-containing cellular metabolic cofactors *in vitro*. In the absence of *in vivo* data we can only hypothesise that non-canonical priming with cofactors occurs in the cells. In support of our hypothesis are the apparent Michaelis constants for the cofactors, which are in the similar range as their cellular concentrations. Michaelis constants measured for ATP, NADH and FAD as initiating substrates for DnaG, were 46.6, 109 and 240 μM, respectively. In actively growing in rich medium *E. coli* cells, concentrations of ATP, NADH and FAD are 9.6 mM, 100–1000 μM and 200 μM, respectively (25,28). These constants for *E. coli* DnaG primase are similar to those measured for *E. coli* RNAP (90 μM for ATP and 380 μM for NADH), which is proven to incorporate non-canonical initiating substrates *in vivo*. Nonetheless, based on these calculations and observations it would be unreasonable to expect a large extent of non-canonical priming, and therefore a strong effect on replication in *E. coli*. Perhaps under specific conditions, such as at the chromosome replication origin, or during replication restart, the non-canonical initiation might become more significant. We can also envisage a higher impact of cofactor-dependent priming in eukaryotes where Okazaki fragments are roughly 10 times shorter due to more frequent initiating events, leading to a higher probability for primase to initiate with cofactors.

Mitochondria is another system where pronounced effects of non-canonical priming on replication may be expected. In mitochondria initiation of replication and transcription are performed by the same enzyme, single stranded mitochondrial RNAP (29–31). It was recently found that mitochondrial RNAP incorporates non-canonical substrates including NAD^+^/NADH and FAD very efficiently both *in vitro* and *in vivo* (19,32).

Despite a presumably modest effect of cofactors on replication *in vivo* in *E. coli*, they may still serve as tools for biochemical and structural studies of a primase. ‘Basic ridge’ amino acid residues specifically influence initiation with cofactors; perhaps this interphase can be further explored to increase the stability of the ternary complex between primase, primer and DNA template, to enable its structure to be determined.

Cofactors might also be useful for understanding the mechanism of inhibition of DnaG primase by ppGpp. We found that non-canonical initiation of RNA synthesis is affected more strongly by ppGpp, i.e. lower concentrations of ppGpp are required to inhibit initiation with cofactors to the same extent as canonical initiation with ATP. The stronger competition between ppGpp and cofactors would lead to early inhibition of the non-canonical priming with growing concentration of ppGpp during onset of stationary phase or starvation. We suggest a mixed type of inhibition by ppGpp for synthesis initiated with any substrate (ATP, NADH and FAD). We suggest that the competitive component of inhibition is more prominent for initiation with cofactors due to a more extensive binding site being shared by ppGpp and cofactors, and by ppGpp interference with basic ridge residues which coordinate non-canonical substrates according to our structural modelling. The non-competitive component of the inhibition might come from ppGpp interference with template binding, as suggested in (15).

This work highlights the differences between RNA polymerases (primase versus transcriptase) utilizing this non-canonical initiation mechanism. Notably, DnaG uses NAD^+^ very inefficiently compared to NADH, in contrast to bacterial and mitochondrial transcriptases, which do not distinguish between the reduced and oxidised forms (19,21). This feature of DnaG might connect priming of replication to the redox state of the cell. We also showed that the template base at the –1 position does not play role in non-canonical substrate utilisation by DnaG, unlike transcriptases, which at least in some instances are sensitive to the identity of the –1 base (20). This result also suggests that the incorporation of cofactors does not lead to spurious initiation.

RNA pieces of Okazaki fragments are destined to be removed. Despite the transient nature of these RNAs, a balance between the kinetics of primer removal and extension influences the mean size and length distribution of Okazaki fragments, and ultimately replication kinetics (11). We showed that the rate of processing of the replication primer by PolI is affected specifically and differentially by the 5′-cofactors; NADH stimulates while FAD inhibits. Therefore, different 5′-cofactors might program for different lifetimes of resulting Okazaki fragments. The extra moiety on the 5′-end of primer might indirectly influence the stability of primase–helicase–primer–DNA complex, similarly to the higher stability of the complex of primase–helicase with DNA in the case of 5′-triphosphorylated primer compared to the unphosphorylated in the bacteriophage T7 system (33). We can also hypothesize that the 5′-cofactor might make primase stall on the DNA template and act as a stronger roadblock, compared to 5′-ATP primer. Indeed it was shown that a primase–primer complex on DNA can act as a roadblock for synthesis of the next Okazaki fragment and leads to replicative polymerase holoenzyme dissociation and recycling in both bacteriophage T4 (34) and *E. coli* systems (35).

Potential primer processing defects caused by cofactors might also lead to increased retention of the short oligoribonucleotides within genomic DNA. These pieces may function as a ribonucleotide imprint, when short RNA regions in DNA serve as epigenetic switches, such as the mating locus switch in yeast, as well as markers for repair machinery recognition to distinguish between old and new DNA strands (36).

Here, we explored the ability of DnaG to incorporate NADH, FAD and DP-CoA cofactors, well established as substrates for RNAPs of transcription. Currently, more potential nucleotide analogues, including dinucleotide polyphosphates incorporated into a 5′-position of cellular RNAs are being discovered in both kingdoms (18). The role of these emerging substrates potentially extends to regulation of initiation of replication.

## Supplementary Material

gkaa447_Supplemental_File
