# Supplementary material for: Metabolic cofactors NADH and FAD act as non-canonical initiating substrates for a primase and affect replication primer processing in vitro
Source: Nucleic Acids Res. 2020 May 28;48(13):7298–306. doi: 10.1093/nar/gkaa447 (PMC7367122; doi:10.1093/nar/gkaa447)
Supplement: gkaa447_Supplemental_File [file gkaa447_supplemental_file.docx]

**Supplementary Information**

**
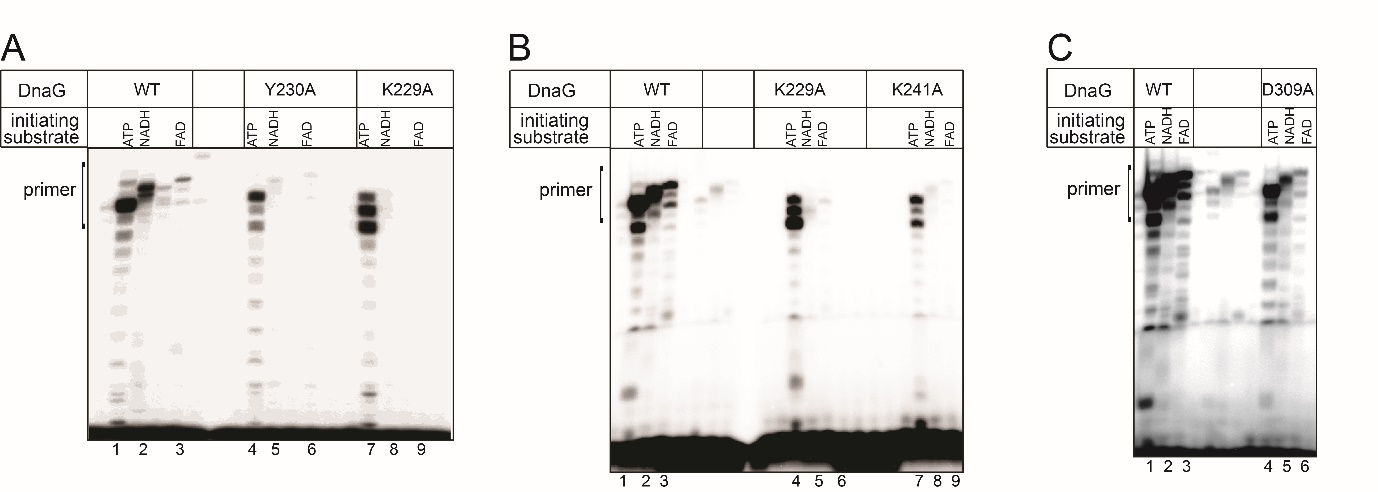
Supplementary Figure 1. Mutants of primase “basic ridge” have specific deficiency in initiating synthesis with NADH and FAD. A,B,C.** Representative gels showing primer synthesis by DnaG primase with indicated amino acid changes using ATP, NADH or FAD as initiating substrate.

**Supplementary Table 1** **– Interactions between nucleotides and basic ridge residues**

|  | **Residue** | **ATP** | **FAD** | **NADH** | **ppGpp** |
| --- | --- | --- | --- | --- | --- |
|  | (atom) | (atom/ distance Å) | | | |
| *E. coli* | K229 (N^𝜻^) | O^γ2^ >4.4 | O^2^ 2 - 2.5 | Oᵟ^2^ 2.5 - 3  N^1N^ 3 – 3.5 | O^1β^ 3.5 – 4 |
| *S. aureus* | K230 (N^𝜻^) | O^1γ^ 2.6 | - | - | O^4’^ 2.6  O^1β^ 3.0 |
| *E. coli* | Y230 (OH) | O^γ1^ 2 – 2.5 | O^4’^ >4 | O5ᵟ >4.5  O4ᵟ >4.5 | O^2β^ 3 – 3.5 |
| *S. aureus* | Y231 (OH) | O^1γ^ 2.6 | - | - | O^2β^ 2.8 |
| *E. coli* | K241 (N^𝜻^) | via H_2_O | via H_2_O  (as ATP) | via H_2_O  (as ATP) | - |
| *S. aureus* | K242 | via H_2_O | - | - | via H_2_O |
| *E. coli* | D309 (Oᵟ^2^) | O^α2^ >3.5 | O^1α^ 3 – 3.5 | O^1α^ 3 – 3.5 | - |
| *S. aureus* | D310 | O^2γ^ 2.9 |  |  | - |

Approximate range of interacting/neighbouring atoms in the *E. coli* modelled complexes is based on the conformation shown in figure 3 C, D, depicting one potential binding mode. For *S. aureus*, distances reported are those in models in PDB.
